# Glucagon‐like peptide‐1 receptor agonist regulates fat browning by altering the gut microbiota and ceramide metabolism

**DOI:** 10.1002/mco2.416

**Published:** 2023-11-20

**Authors:** Ke Lin, Chunyan Dong, Binyan Zhao, Bailing Zhou, Li Yang

**Affiliations:** ^1^ Department of Biotherapy, Cancer Center and State Key Laboratory of Biotherapy West China Hospital Sichuan University Chengdu China

**Keywords:** ceramide, GLP‐1 RA, gut microbiota, *L. reuteri*, obesity

## Abstract

Studies have shown that antidiabetic drugs can alter the gut microbiota. The hypoglycemic effects of the drugs can be attributed in part to certain species in the gut microbiome that help the drugs work more effectively. In addition, increasing energy expenditure via the induction of adipose tissue browning has become an appealing strategy to treat obesity and associated metabolic complications. Currently, glucagon‐like peptide‐1 receptor agonist (GLP‐1 RA) treatment for metabolic disorders such as obesity and type 2 diabetes has been widely studied. To determine the mechanism of a long‐acting GLP‐1 RA affects adipose tissue browning and the gut microbiome, we treated high‐fat diet mice with GLP‐1 RA and demonstrated that the drug can regulate adipose tissue browning. 16S rRNA and untargeted metabolomics assays suggested that it increased the abundance of bacterium *Lactobacillus reuteri* and decreased serum ceramide levels in mice. *L. reuteri* was negatively correlated with ceramide. We found that the mechanism of ceramide decline was alkaline ceramidase 2 (Acer2) overexpression. Moreover, *L. reuteri* can play a therapeutic synergistic role with GLP‐1 RA, suggesting that gut microbiota can be used as a part of the treatment of diabetes.

## Graphical abstract

GLP‐1 RA can reduce body weight and regulate adipose tissue browning in high‐fat diet mice. 16S rRNA and untargeted metabolomics assays suggested that it reshapes the gut microbiome and influences the metabolite ceramide in mice by upregulating Acer2 expression, and *L. reuteri* was negatively correlated with ceramide. Moreover, *L. reuteri* can play a therapeutic synergistic role with GLP‐1 RA.

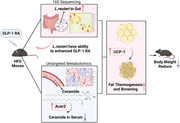

## 1 INTRODUCTION

Obesity is a major health problem, with its incidence increasing dramatically in both industrialized and developing countries.^1^, ^2^ Obesity is the result of a long‐term imbalance between energy intake and expenditure due to an excessive increase in body fat. Obesity predisposes individuals to a variety of diseases, including diabetes, cardiovascular disease, nonalcoholic fatty liver disease, cancer, and some immune‐related diseases.^3^, ^4^, ^5^

The main characteristic of obesity is an increase in adipose tissue throughout the body. Mammalian adipose tissue can be divided into white adipose tissue (WAT), brown adipose tissue (BAT), and beige adipose tissue according to its function and morphology. WAT is the most common adipose tissue, and the accumulation of WAT is one of the manifestations of obesity. The main difference between WAT and BAT is that BAT has more mitochondria and small fat droplets, whereas WAT has larger fat droplets and fewer mitochondria.^6^ BAT plays a role in thermogenesis, generating heat through the combustion of unconjugated protein‐1 (UCP‐1) with ATP to produce unconjugated nutrients.^7^ Beige adipose tissue has been identified as an intermediate between BAT and WAT, which has the thermogenic capacity and morphological characteristics of BAT. WAT into beige adipose tissue is known as fat browning. Fat browning has been implicated in the treatment and prevention of type 2 diabetes (T2D) and other common metabolic disorders. Studies have found that BAT takes glucose and lipids from the blood circulation, helps clear glucose, and reduces the demand for insulin secretion by beta cells, thereby improving islet function.^8^

Although the root cause of obesity is excessive caloric intake, differences in gut microbiota between individuals may be important factors affecting energy homeostasis. The human gut is mainly composed of *Bacteroidetes* and *Firmicutes*.^9^ Studies have shown that the ratio of *Bacteroidetes*/*Firmicutes* in obese individuals is small relative to that in lean individuals, and when obese individuals are subjected to fat restriction or carbohydrate restriction, the relative abundance of *Bacteroidetes* increases and the abundance of *Firmicutes* decreases.^10^ Patients with T2D had moderate levels of gut bacterial dysregulation, a decrease in bacteria producing metabolically beneficial butyrate, and an increase in several pathogens.^11^ Antidiabetic drugs can alter the gut microbiota when treating T2D.^12^ Liraglutide, metformin, and glitazone have been shown to alter the gut microbiota.^12^, ^13^, ^14^, ^15^ As research on the effects of these drugs on the gut microbiota continues to grow, the hypoglycemic effects of the drugs can be attributed in part to certain species in the gut microbiome that help the drugs work more effectively.^12^ Recent studies have also linked obesity to a variety of specific bacteria, such as *Akkermansia muciniphila*, *Bifidobacteria*, and *Lactobacillus*. Several independent trials have shown that supplementation with *A. muciniphila* can significantly improve metabolism, increase insulin sensitivity, and reduce plasma triglycerides.^16^, ^17^ Similarly, *Lactobacillus* and *Bifidobacteria* also play an important role in the microecological balance of the human gut tract. For example, *Bifidobacteria* has been used to treat obese youth with marked improvement in insulin sensitivity.^18^

Diseases caused by obesity are caused in part by the abnormal accumulation of harmful lipid metabolites in tissues such as the liver and heart. Among these lipid metabolites, ceramides and other sphingolipids attract attention, which can cause insulin resistance and dyslipidemia.^19^ Gut microbiota also regulates ceramide metabolism.^20^ However, few studies have explored the relationship between ceramide metabolism and glucagon‐like peptide‐1 receptor agonist (GLP‐1 RA).

Currently, GLP‐1 is an important target for the treatment of T2D and obesity.^21^ (Ex‐4)_2_‐Fc is a long‐acting GLP‐1 RA developed in our laboratory that is fused by Exendin‐4 with the Fc fragment mutant of human immunoglobulin IgG4.^22^, ^23^ In this study, the effects of GLP‐1 RA on adipose thermogenesis and browning were verified, and 16S gene sequencing of the gut complex metabolic network and nontargeted metabolomics studies were comprehensively analyzed to explore the mechanism of GLP‐1 RAs in obese mice.

## 2 RESULTS

### 2.1 GLP‐1 RA improves obesity and promotes fat browning in HFD mice

**FIGURE 1:**
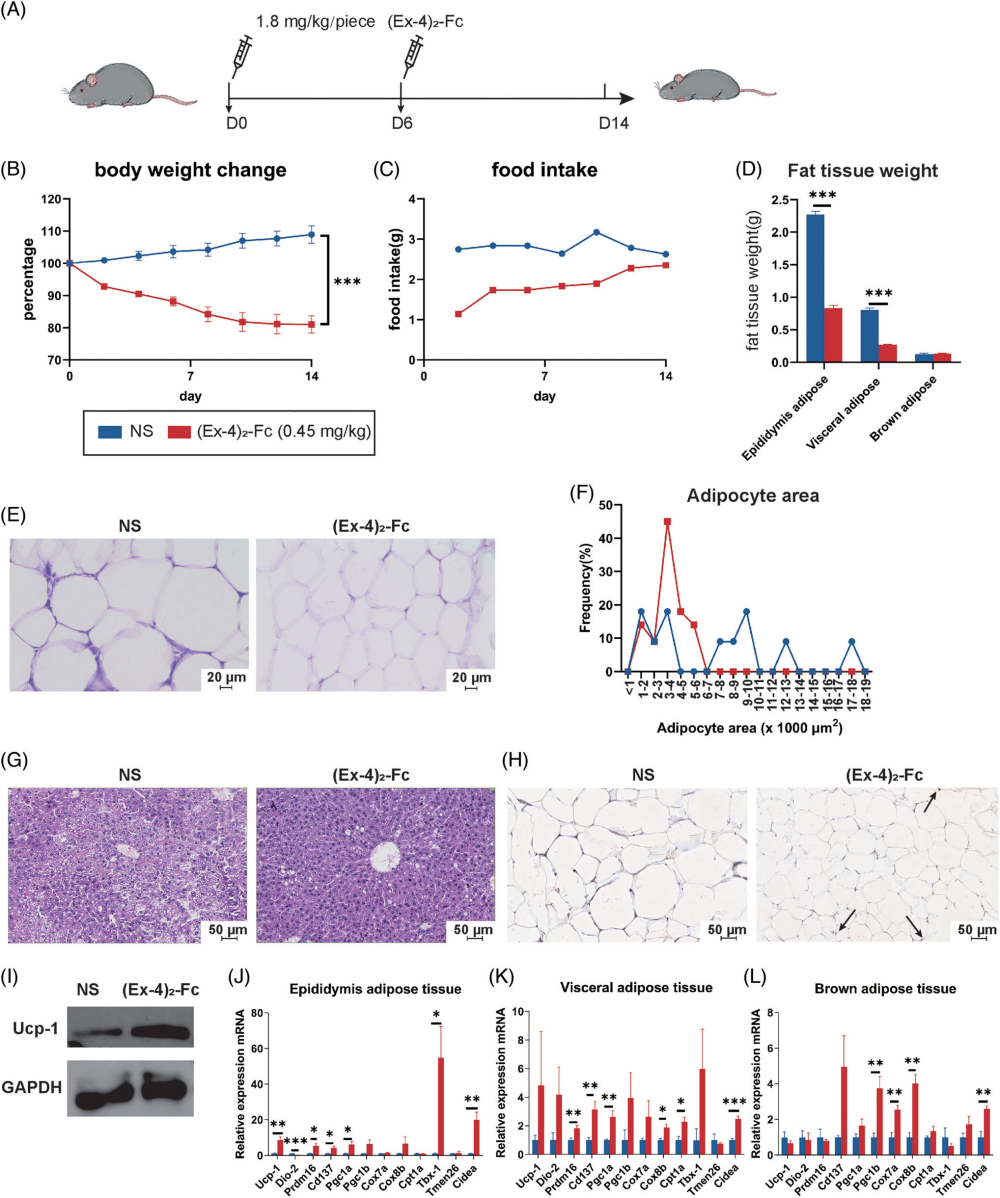
Glucagon‐like peptide‐1 receptor agonist (GLP‐1 RA) improves obesity and fat browning in HFD mice: (A) HFD mice were treated with GLP‐1 RA once every 6 days for 2 weeks; (B) changes in the body weight of mice; (C) average daily food intake of mice; (D) weight of fat adipose tissue; (E) epididymis adipose tissue hematoxylin and eosin (H&E) staining. Bar = 20 μm; (F) adipocyte area (μm^2^) distribution; (G) H&E staining of representative liver sections. The arrow points to the unconjugated protein‐1 (UCP‐1) protein. Bar = 50 μm; (H) immunohistochemical observation of UCP‐1 in epididymis adipose tissue. Bar = 50 μm; (I) Western blot of UCP‐1; (J–L) mRNA expression of thermogenic genes such as UCP‐1, Dio‐2, Pram16, Cd137, and Cox8b increased in three adipose tissues. Data are expressed as the mean ± standard error. Data are shown as the mean ± SEM. *p < 0.05, **p < 0.01, and ***p < 0.001 versus the NS group.

First, to verify the efficacy of GLP‐1 RA in improving obesity, HFD mice were treated with 1.8 mg/kg (Ex‐4)_2_‐Fc once every 6 days for 2 weeks (Figure 1A). Compared with the NS group, the body weight of mice in the (Ex‐4)_2_‐Fc group decreased significantly (Figure S1A), with a weight loss of approximately 19% (Figure 1B). In addition, through food intake monitoring, it was proven that the average daily food intake of mice decreased significantly, but with the passage of time, the average daily food intake of mice gradually increased (Figure 1C). After (Ex‐4)_2_‐Fc treatment, the weight of epididymis adipose tissue and visceral adipose tissue of mice decreased significantly, whereas there was no significant difference in BAT (Figure 1D). Hematoxylin and eosin (H&E) staining showed (Figure 1E) that the adipocyte area distribution in the NS group was larger than that in the (Ex‐4)_2_‐Fc group (Figure 1F). Liver H&E staining showed (Figure 1G) that the arrangement of liver cells in the NS group was disordered with vacuole‐like degeneration of varying sizes, whereas the liver cells in the (Ex‐4)_2_‐Fc group were arranged radially around the central vein, with clear structure, normal morphological arrangement, and uniform cytoplasm. In conclusion, GLP‐1 RA can significantly reduce the body weight of mice and reduce liver injury caused by a high‐fat diet.

To understand the changes in adipose tissue browning and thermogenesis in mice treated with (Ex‐4)_2_‐Fc, qPCR was performed on epididymis adipose tissue, visceral adipose tissue, and BAT. The results are shown in the figure. Generally, after (Ex‐4)_2_‐Fc treatment, the mRNA expression of thermogenic genes, such as UCP‐1, Dio‐2, Pram16, Cd137, and Cox8b, increased in three adipose tissues (Figure 1J–L), among which the adipose tissue of the epididymis had the most obvious changes. Immunohistochemistry and Western blot observation of UCP‐1 in epididymis adipose tissue showed an increased expression of UCP‐1 protein (Figure 1H,I). These results indicate that (Ex‐4)_2_‐Fc can enhance the browning and thermogenesis of adipose tissue.

### 2.2 GLP‐1 RA changes gut microbiota

**FIGURE 2:**
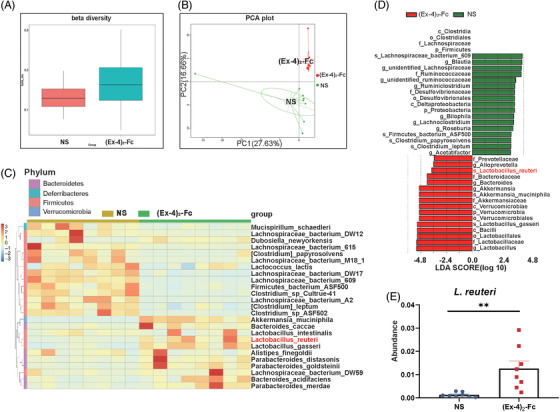
Glucagon‐like peptide‐1 receptor agonist (GLP‐1 RA) changes gut microbiota: (A) beta diversity; (B) principal component analysis (PCA) diagram; (C) heatmap of species clustering; (D) LefSe analysis; (E) abundance of *Lactobacillus reuteri*. *n* = 8. Data are shown as the mean ± SEM. *p < 0.05, **p < 0.01 and ***p < 0.001 versus NS group.

To explore the mechanism of (Ex‐4)_2_‐Fc treatment in obese mice from the perspective of gut microbiota, mouse feces were collected after the treatment cycle, and 16S sequencing was performed. Overall, gut microbiota composition changed after (Ex‐4)_2_‐Fc treatment (Figure S1B), and beta diversity increased (*p* = 0.0256; Figure 2A), indicating increased differences in species composition. However, there was no significant difference in alpha diversity (expressed as the ACE index) after (Ex‐4)_2_‐Fc treatment (*p* = 0.0537; Figure S1C), indicating no significant change in overall species richness after (Ex‐4)_2_‐Fc treatment. Principal component analysis (PCA) directly showed the difference in gut microbiota structure between the two groups (Figure 2B; PC1 was 27.63%, and PC2 was 16.66%), indicating that the symbols of mice in the NS group were distributed in the second quadrant, whereas those in the (Ex‐4)_2_‐Fc group were mainly distributed in the third quadrant. The above results indicated that the gut microbiota structure and composition of mice were significantly changed after treatment with (Ex‐4)_2_‐Fc. The top 25 species with the highest abundance were selected for clustering from the two levels of species and samples, and a heatmap was drawn (Figure 2C). After (Ex‐4)_2_‐Fc treatment, *bacteroides_caccae*, *Alistipes_finegoldii*, *Parabacteroides_distasonis*, *Parabacteroides_goldsteinii*, *Bacteroides_acidifaciens*, *Parabacteroides_merdae*, *Akkermansia_muciniphila*, *Lactobacillus_inteslinalis*, *Lactobacillus_reuteri*, and *Lactobacillus_distasonis* increased. To find statistically significant biological markers (biomarker), LefSe analysis was performed on gut microbiota (Figure 2D). At the species level, *Lactobacillus reuteri* was enriched after (Ex‐4)_2_‐Fc treatment (Figure 2E), which has been linked in previous studies to reducing obesity and improving metabolism.^24^ These results indicated that (Ex‐4)_2_‐Fc treatment reshaped the intestinal flora of mice and enriched the *L. reuteri* level in the intestine.

### 2.3 *L. ruteri* enhances the weight‐loss effect of GLP‐1 RA

**FIGURE 3:**
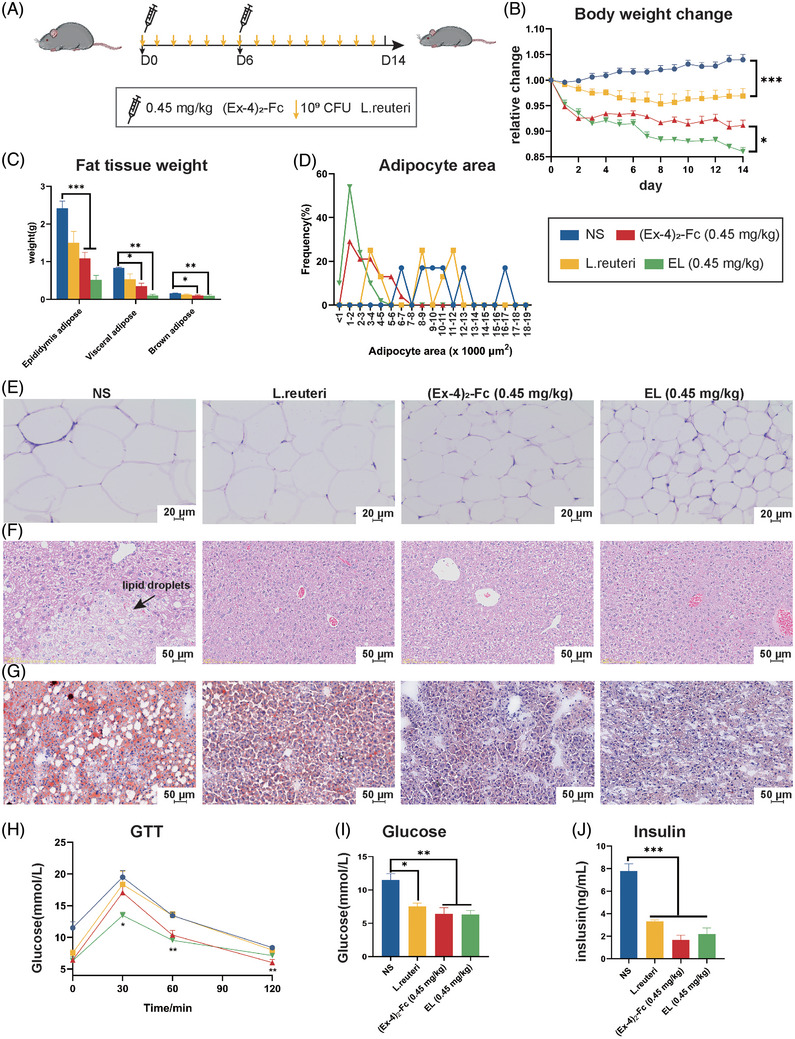
*Lactobacillus reuteri* enhanced the weight‐loss effect of glucagon‐like peptide‐1 receptor agonist (GLP‐1 RA): (A) HFD mice were treated with GLP‐1 RA (0.45 mg/kg) once every 6 days and *L. reuteri* every day; (B) changes in the body weight of mice. *L. reuteri* versus NS group and EL versus (Ex‐4)_2_‐Fc group; (C) weight of fat adipose tissue; (D) adipocyte area (μm^2^) distribution; (E) epididymis adipose tissue hematoxylin and eosin (H&E) staining. Bar = 20 μm; (F) H&E staining of representative liver sections. Bar = 50 μm; (G) oil red O staining of representative liver sections. Bar = 50 μm; (H) GTT in mice; (I) mice fasting blood glucose; (J) mice fasting blood insulin. Data are expressed as the mean ± standard error. Data are shown as the mean ± SEM. *p < 0.05, **p < 0.01 and ***p < 0.001.

To investigate the role of *L. reuteri* in (Ex‐4)_2_‐Fc weight loss, *L. reuteri* was combined with different doses of (Ex‐4)_2_‐Fc (1.8, 0.9, and 0.45 mg/kg), (Ex‐4)_2_‐Fc was administered once every 6 days for 2 weeks (Figure S1D–F). EL group was treat with (Ex‐4)_2_‐Fc and *L. reuteri* simultaneously. Compared with the (Ex‐4)_2_‐Fc group, the *L. reuteri* combined with (Ex‐4)_2_‐Fc group had significantly reduced body weight (Figure S1D–F). When the dose was reduced to 0.45 mg/kg, the weight change was statistically significant (Figure 3A,B and Figure S1D). These results indicated that when the dose of (Ex‐4)_2_‐Fc was reduced to 0.45 mg/kg, the body weight difference between the (Ex‐4)_2_‐Fc group, and EL group was the most significant. In addition, both *L. reuteri* and (Ex‐4)_2_‐Fc reduced food intake in mice, with the lowest intake on the first day of treatment and then increased with the number of days of treatment (Figures S1G). Weighing (Figure 3C) and H&E staining of adipose tissue of the epididymis (Figure 3E) showed that the adipose–tissue weight, adipocyte area in the EL (0.45 mg/kg) group were significantly lower than those in the (Ex‐4)_2_‐Fc (0.45 mg/kg) group (Figure 3D).

Compared with NS mice, *L. reuteri* and (Ex‐4)_2_‐Fc treatment liver tissue cells were arranged neatly (Figure 3F) and reduced the number and size of lipid droplets (Figure 3G). Glucose tolerance test results showed that the EL (0.45 mg/kg) group had significantly lower blood sugar levels than the NS group at 30, 60, and 120 min (Figure 3H). At 60 min, blood glucose in the EL (0.45 mg/kg) group was also lower than that in the NS group. The comparison of area AUC under the GTT curve in 0–120 min showed that, compared with the NS group, there were statistically significant differences among the three groups (Figure S1H,I). These results suggest that *L. reuteri* can enhance the ability of low‐dose (Ex‐4)_2_‐Fc to improve glucose tolerance. HFD mice are characterized by hyperglycemia and hyperinsulinemia. After treatment, fasting blood samples were taken to measure glucose and insulin levels. The blood glucose and insulin levels of the three treatment groups were lower than those of the NS group, and there were significant differences (Figure 3I,J). However, there was no significant difference in blood glucose content and insulin content between the (Ex‐4)_2_‐Fc (0.45 mg/kg) group and the EL (0.45 mg/kg) group. These results suggest that *L. reuteri* alone can reduce blood glucose and insulin content in HFD mice, but it is not significant to enhance the efficacy of low‐dose GLP‐1 RA. In general, the combination of (Ex‐4)_2_‐Fc and *L. reuteri* can improve obesity, liver damage, and metabolism, especially when combined with a low dose of (Ex‐4)_2_‐Fc.

### 2.4 *L. reuteri* enhances fat browning effect of GLP‐1 RA

**FIGURE 4:**
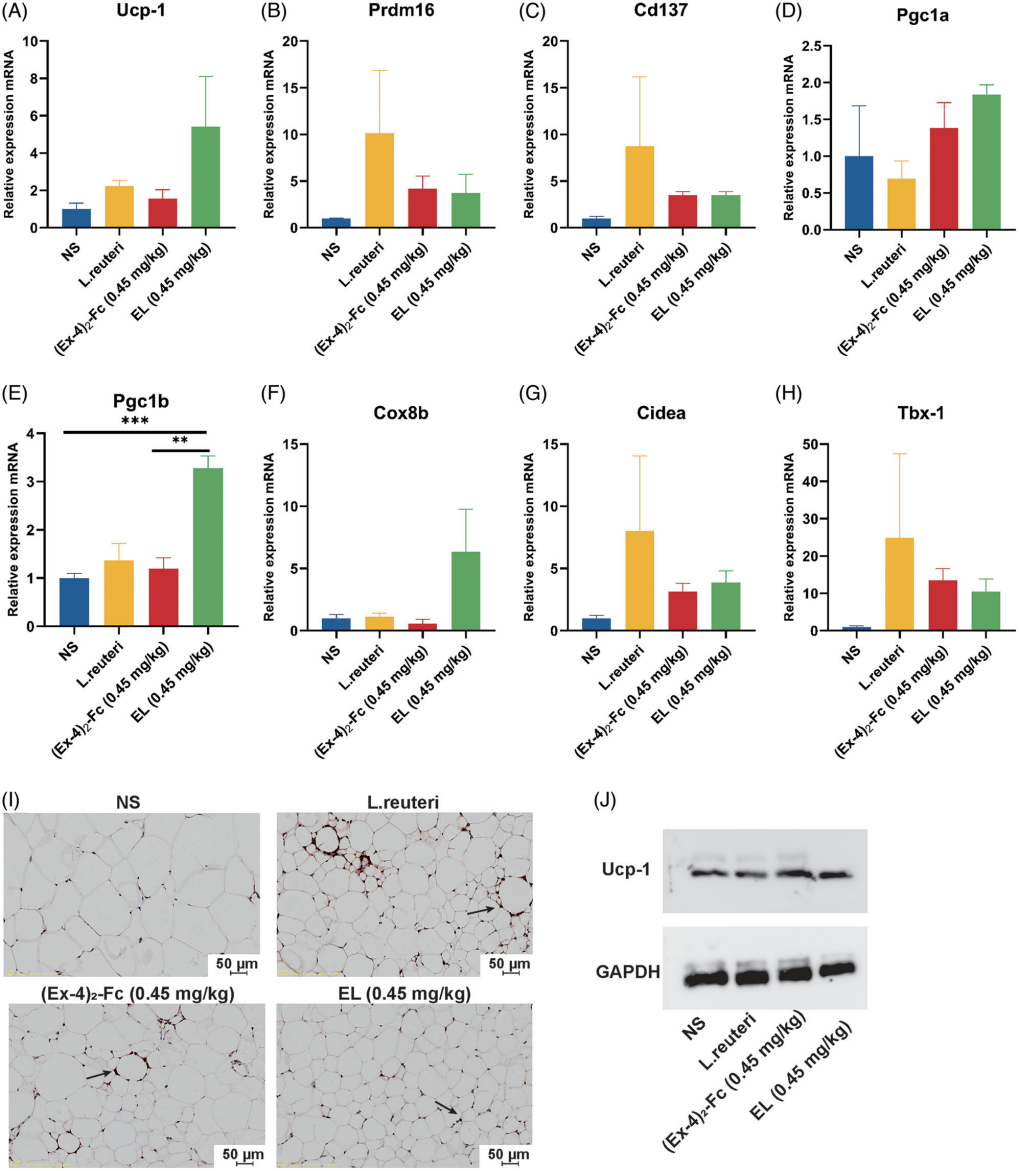
*Lactobacillus reuteri* enhanced fat browning: (A–I) relative mRNA expression of unconjugated protein‐1 (UCP‐1), Prdm16, Cd137, Pgc1a, Pgc1b, Cox8b, Cidea, and Tbx‐1. (I) UCP‐1 immunohistochemical section of epididymis adipose tissue. The arrow points to the UCP‐1 protein; (J) Western blot of UCP‐1. Data are shown as the mean ± SEM. *p < 0.05, **p < 0.01 and ***p < 0.001 versus the NS group. Bar = 50 μm.

To investigate whether *L. reuteri* could enhance the effect of low‐dose GLP‐1 RA on adipose thermogenesis and browning, mouse epididymis adipose tissue was collected, and the expression of thermogenesis genes was detected. The results showed that 0.45 mg/kg (Ex‐4)_2_‐Fc could increase the expression of UCP‐1, Prdm16, Cd137, Pgc1a, Pgc1b, Cox‐8b, Tbx‐1, and Cidea (Figure 4A–H). Immunohistochemical biopsy (Figure 4I) and Western blot (Figure 4J) results showed that compared with the NS group, UCP‐1 expression in adipose tissue of *the L. reuteri* group, (Ex‐4)_2_‐Fc (0.45 mg/kg), group and EL (0.45 mg/kg) group was increased. These results indicate that *L. reuteri* can increase the thermogenesis of fat, and combined with (Ex‐4)_2_‐Fc treatment, it can also enhance the thermogenesis of fat.

### 2.5 GLP‐1 RA changes lipid metabolism

**FIGURE 5:**
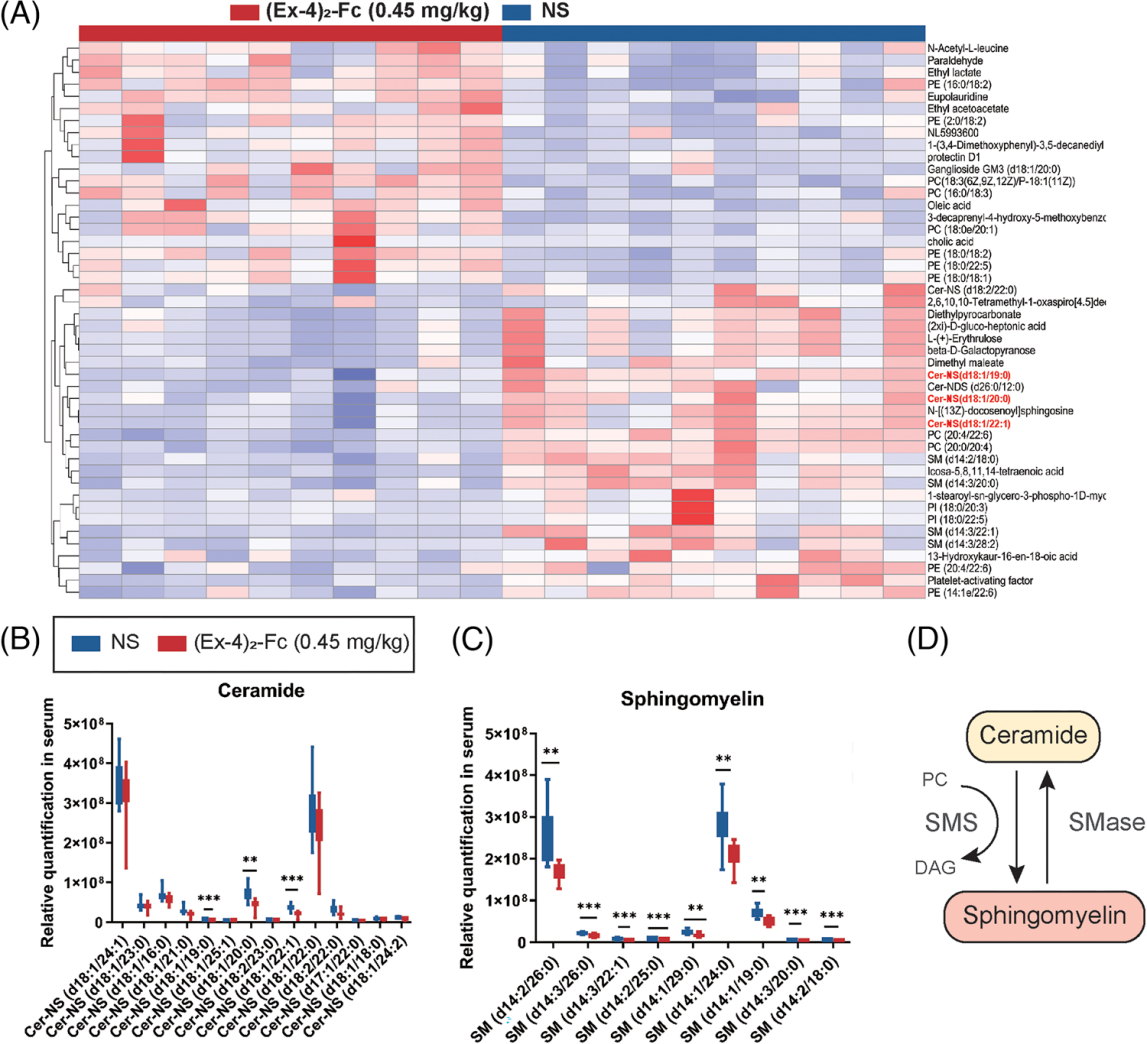
Metabolic changes after glucagon‐like peptide‐1 receptor agonist (GLP‐1 RA) treatment: (A) nonclustering heatmap of negative ion differential metabolites; (B) serum ceramide relative expression level; (C) relative expression level of serum sphingomyelin; (D) ceramide and sphingomyelin are converted under sphingomyelin synthase (SMS) and sphingomuelinase (SMase). Data are shown as the mean ± SEM. **p < 0.01 and ***p < 0.001 versus the NS group.

To explore the effects of GLP‐1 RA on lipid metabolism in HFD mice, a lipid omics study was conducted on the serum of 1.8 mg/kg (Ex‐4)_2_‐Fc‐treated mice. PCA was used to observe the overall distribution trend between the two groups of samples, and the results showed that the serum lipid composition of (Ex‐4)_2_‐Fc‐treated mice significantly changed (Figure S2A,B; PC1 = 24.82%, PC2 = 14.67%; negative ion PC1 = 21.02%, PC2 = 11.67%). According to VIP, FC, and *p* value parameters, 106 of the positive lipid compounds were changed. Compared with the NS group, 40 of them were upregulated and 66 of them were downregulated. These results indicate that 1.8 mg/kg (Ex‐4)_2_‐Fc can significantly change the overall lipid metabolism of HFD mice. To further explore the differences in the composition of lipid compounds, the relative quantitative values of differential metabolites obtained from samples of the two groups were normalized and converted to obtain the differences in metabolic expression patterns between and within the same comparison pair between the two groups, and the nonclustering heatmap of differential metabolites was obtained (Figure 5A). Changes in lipid metabolite composition in samples after treatment include changes in sphingolipids, such as phosphatidylcholine (PC), phosphatidylinositol (PI), phosphatidylethanolamine (PE), sphingomyelin (SM), and ceramide (Cer). Ceramide has been linked to obesity and metabolic disorders and has been shown to regulate adipose browning, inflammation, and metabolism. Analysis of the relative changes in ceramide in serum showed that after 1.8 mg/kg (Ex‐4)_2_‐Fc treatment, ceramide decreased significantly in the samples (Figures 5B). Sphingomyelin and ceramide can be converted into each other in the cell (Figure 5C). The results showed that sphingomyelin was also decreased (Figure 5D). These results indicate that (Ex‐4)_2_‐Fc treatment can significantly influence the lipid metabolism of HFD mice, especially reducing ceramide level.

### 2.6 Ceramides are negatively correlated with *Lactobacillus*

**FIGURE 6:**
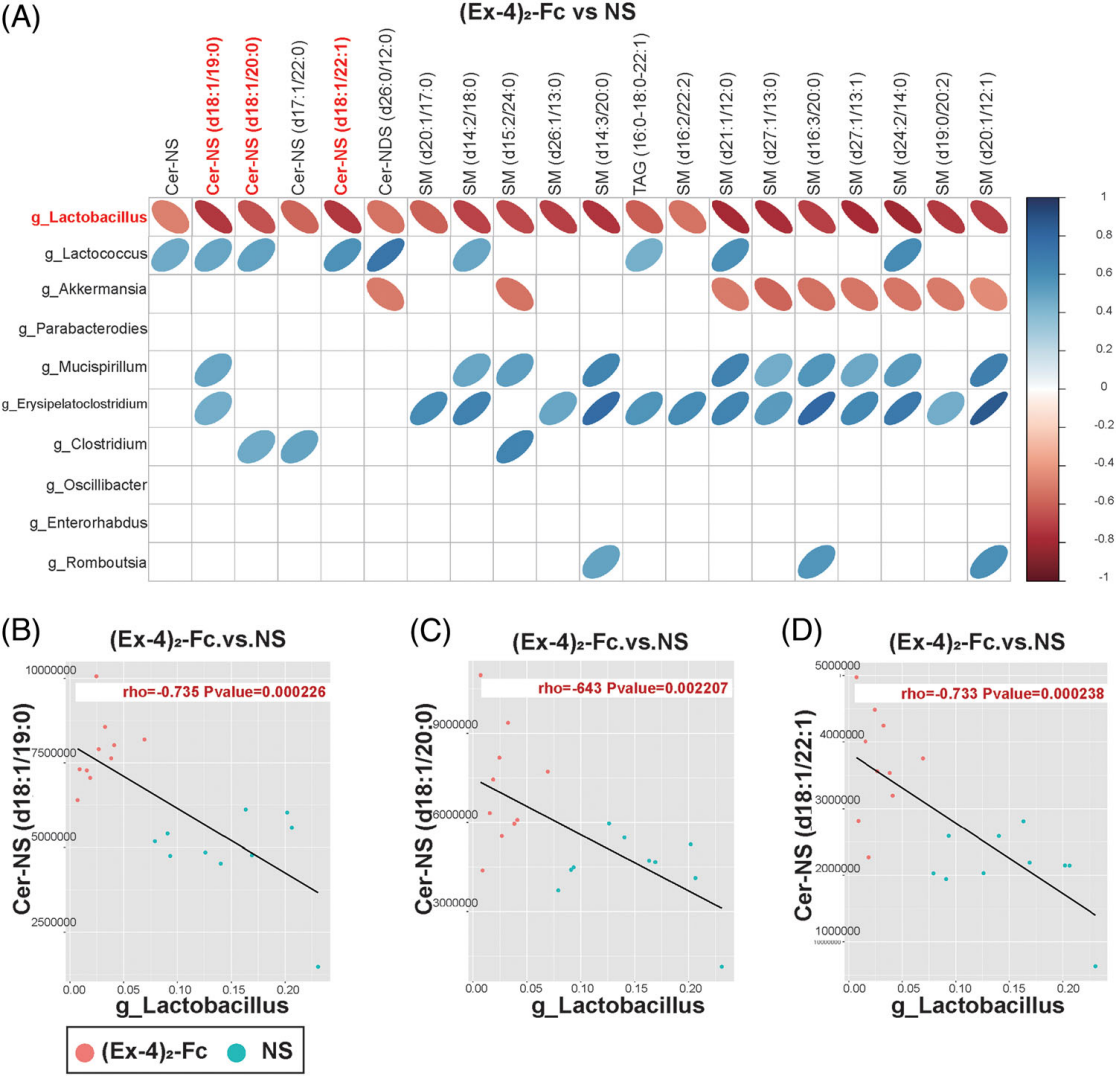
Negative correlation between ceramide content and *Lactobacillus* level: (A) heatmap of the correlation between differential bacteria and differential metabolite expression. The closer the circle is to the ellipse, the greater the absolute value of correlation. Left‐leaning is negatively correlated, right‐leaning is positively correlated; (B) scatter plot of Cer‐NS (d18:1/19:0) correlation with *Lactobacillus*; (C) scatter plot of Cer‐NS (d18:1/20:0) correlation with *Lactobacillus*; (D) scatter plot of Cer‐NS (d18:1/22:1) correlation with *Lactobacillus*.

To clarify the relationship between the gut microbiota and metabolites, we conducted correlation analysis between bacteria with significant differences at the genus level obtained by 16S rDNA analysis of the NS and (Ex‐4)_2_‐Fc groups and metabolomics analysis of significantly different metabolites to measure the degree of association between species diversity and metabolites in samples. Heatmap analysis of the correlation between differential bacteria and differential metabolite expression showed that *Lactobacillus* (*Lactobacillus gasseri*, *L. reuteri*, and *Ligula intestinalis*) was negatively correlated with the top 20 differential metabolites (correlation coefficient was less than 0). Three ceramides and six sphingomyelins were negatively associated with *Lactobacillus* (Figure 6A). Further analysis of the *Lactobacillus* and ceramide correlation scatter diagram was performed, the Pearson correlation coefficient of rho and *p* value was calculated, and | rho | 0.6 or higher was selected. At the same time, *p* < 0.05 or less resulted in scatter plot analysis. Cer‐NS (d18:1/19:0), Cer‐NS (d18:1/20:0), and Cer‐NS (d18:1/22:1) in ceramides were negatively correlated with *Lactobacillus* (Figure 6B–D). Similarly, sphingomyelin was also negatively correlated with *Lactobacillus* (Figure S2C–H).

### 2.7 GLP‐1 RA enhances ceramide metabolism through Acer2

**FIGURE 7:**
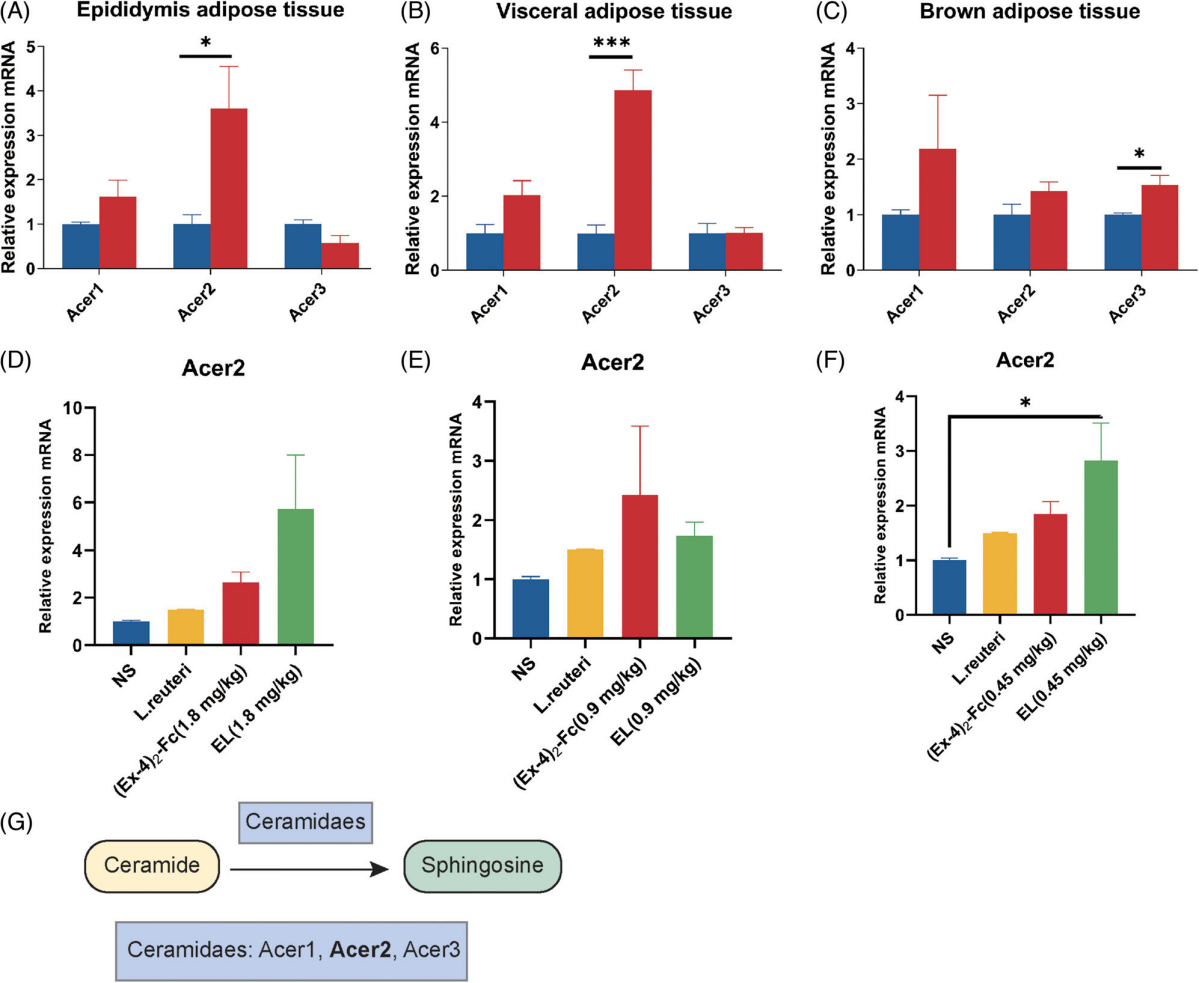
Glucagon‐like peptide‐1 receptor agonist (GLP‐1 RA) enhances ceramide metabolism through alkaline ceramidase 2 (Acer2): (A) relative expression level of alkaline ceramidase mRNA in epididymis adipose tissue; (B) relative expression level of alkaline ceramidase mRNA in visceral adipose tissue; (C) relative expression level of alkaline ceramidase mRNA in brown adipose tissue; (D) relative expression level of Acer2 mRNA in epididymis adipose tissue treated with 1.8 mg/kg (Ex‐4)_2_‐Fc; (E) relative expression level of Acer2 mRNA in epididymis adipose tissue treated with 0.9 mg/kg (Ex‐4)_2_‐Fc; (F) relative expression level of Acer2 mRNA in epididymis adipose tissue treated with 0.45 mg/kg (Ex‐4)_2_‐Fc; (G) ceramides are metabolized to sphingosine by ceramidaes. Data are shown as the mean ± SEM. *p < 0.05 and ***p < 0.001 versus the NS group.

Ceramide synthesis and metabolism involve three pathways: the de novo pathway, sphingomyelin hydrolysis, and remediation pathways.^25^ Therefore, to explore the mechanism by which (Ex‐4)_2_‐Fc reduces ceramide levels, mRNA expression of ceramide synthetase (de facto synthesis pathway) in adipose tissue of 1.8 mg/kg (Ex‐4)_2_‐Fc HFD mice was detected. Interestingly, the results showed an increased expression of genes involved in de novo ceramide synthesis in all three adipose tissues (Figure S2I–K). To explore the molecular mechanism of the reduced ceramide level, alkaline ceramide (Acer) enzymes’ gene expression was examined. The expression of Acer1 and Acer2 increased in adipose epididymis tissue with a significant difference (Figure 7A), the expression of Acer1 and Acer2 increased in visceral adipose tissue, and the expression of Acer2 was significantly different (Figure 7B). Acer3 did not change significantly in epididymis adipose tissue and visceral adipose tissue but increased in BAT (Figure 7C). The above results show that (Ex‐4)_2_‐Fc can increase Acer2 expression in adipose tissue and strengthen the metabolism of ceramide, thereby reducing the levels of serum ceramide. To explore whether *L. reuteri* enhances the metabolism of ceramide in the process of GLP‐1 RA treatment. Therefore, to determine whether *L. reuteri* supplementation can increase the expression level of Acer2 mRNA in the epididymis fat of HFD mice, qPCR results showed that, consistent with previous results, (Ex‐4)_2_‐Fc increased Acer2 expression regardless of the dose (Figure 7D–F). Compared with the NS group, Acer2 expression was increased, which may promote ceramide metabolic degradation. When combined with (Ex‐4)_2_‐Fc, the relative expression level of Acer2 mRNA in the EL (1.8 and 0.45 mg/kg) groups was increased. In conclusion, (Ex‐4)_2_‐Fc reduces ceramide level through Acer2 overexpression, and *L. reuteri* may play role in the progress (Figure 7G).

## 3 DISCUSSION

GLP‐1 RAs are a type of new T2D drug that simultaneously reduces appetite and expected food consumption and increases feelings of satiety and abdominal fullness. All GLP‐1 RAs, after long‐term treatment, can lead to varying degrees of weight loss.^26^ Due to GLP‐1 RAs’ potential weight management capabilities, fat and other GLP‐1 RAs are approved for the treatment of obesity.^27^, ^28^, ^29^ The (Ex‐4)_2_‐Fc used in this study is a stable and long‐acting GLP‐1 RA that can effectively reduce the body weight and food intake of HFD mice.^22^ Studies have shown that the gut microbiota is involved in regulating various metabolic pathways in the host body, including energy homeostasis, glucose metabolism, and lipid metabolism.^30^

To explore the mechanism by which GLP‐1 RA reduces body weight in mice from the perspective of gut microbiota, we established a HFD mouse model to verify the effect of GLP‐1 RA. Based on a comprehensive analysis of their gut microbiota and lipid metabolism data, we identified *L. reuteri* and ceramide as one of the possible mechanisms by which GLP‐1 RA helps reduce body weight in HFD mice. First, consistent with previous results,^22^ (Ex‐4)_2_‐Fc significantly reduced body weight and food intake, decreased adipocyte diameter and area, and improved hepatic steatosis in HFD mice compared with controls. In addition, after (Ex‐4)_2_‐Fc treatment, the expression of middle heat and browning genes was increased in the adipose tissue of mice, indicating that adipose browning occurred in the WAT of HFD mice after treatment, and overall heat production was increased. This will help relieve obesity and blood sugar and improve insulin sensitivity.^31^ Adipose thermogenesis is essential for adaptation to cold environments in mice and humans.^32^ In addition, increased adipose thermogenic activity in mice protects against weight gain and metabolic dysfunction.^33^ 16S gut microbiota analysis data showed that (Ex‐4)_2_‐Fc changed the gut microbiota structure of HFD mice, and the difference in species composition became larger. Further LefSe analysis was performed to search for biomarkers, and *L. reuteri* was found to be enriched after treatment. Many previous studies have shown that *L. reuteri* is an important probiotic that can control weight and obesity, improve insulin sensitivity and glucose homeostasis, increase gut integrity and immune regulation, and reduce liver disease.^2^

To investigate whether *L. reuteri* in (Ex‐4)_2_‐Fc plays a role in the treatment of HFD mice, different doses of (Ex‐4)_2_‐Fc were combined with *L. reuteri*. The results showed that *L. reuteri* alone could control body weight and food intake, improve liver lipid deposition, improve metabolism, and increase fat thermogenesis in HFD mice. In combination with (Ex‐4)_2_‐Fc treatment, the effect was more significant. Interestingly, at different doses of (Ex‐4)_2_‐Fc combined with *L. reuteri*, we found that the lower the dose of (Ex‐4)_2_‐Fc, the greater the difference in weight change compared with the EL group, possibly because the normal dose of (Ex‐4)_2_‐Fc had already reached the limit of weight loss in mice. After reducing the dose of (Ex‐4)_2_‐Fc, *L. reuteri* could enhance the ability of (Ex‐4)_2_‐Fc to control body weight and food intake, improve metabolism, and enhance fat heat production.

As obesity is closely related to metabolism and the gut microbiota often affects host physiological function by influencing body metabolism, lipid omics analysis was performed on (Ex‐4)_2_‐Fc‐treated HFD mice. By lipomic analysis, we found that (Ex‐4)_2_‐Fc can also significantly change ceramide level after treatment. Sphingolipids, ceramides in particular, are thought to be associated with obesity and metabolic disorders and are markers of metabolic diseases, such as insulin resistance, diabetes, and cardiovascular disease.^34^, ^35^, ^36^, ^37^ Reducing ceramide levels in rodents, including blocking ceramide synthesis and increasing ceramide metabolism, reduces insulin resistance and improves diabetes,^38^, ^39^ dyslipidemia and liver steatosis,^40^, ^41^ and cardiovascular disease.^42^ The mechanisms of ceramide action in obesity and metabolic disorders are inhibition of insulin signaling,^19^ reduction of fat thermogenesis and browning,^43^ and inflammatory response.^44^ However, ceramide and sphingomyelin levels decreased significantly after (Ex‐4)_2_‐Fc treatment, suggesting that one of the mechanisms of (Ex‐4)_2_‐Fc weight loss is a decrease in circulating ceramide. Then, to clarify the relationship between gut microbiota and lipid metabolites, we conducted correlation analysis and found that ceramide was negatively correlated with *Lactobacillus*.

To explore the mechanism by which (Ex‐4)_2_‐Fc affects ceramide metabolism, the enzyme of ceramide metabolism was studied. Ceramide metabolism involves three pathways: the de novo pathway, sphingomyelin hydrolysis, and remediation.^25^ Interestingly, however, the expression of genes such as Spt (serine palmitoyl) and CerS (ceramide synthase), which are responsible for de novo ceramide synthesis, was enhanced in adipose tissue, which may be related to the feedback regulation of ceramide. Our study found that the expression of Acer2, a ceramide alkaline metabolizing enzyme, was significantly increased after (Ex‐4)_2_‐Fc treatment and tended to increase after combined treatment with *L. reuteri*. In addition, a study found that the adipose overexpression of Acer2 rescued adipocyte HIF‐2α‐deficiency‐induced exacerbation of atherosclerosis. Furthermore, it had protective effects on atherosclerosis, accompanied by a reduction in adipose and plasma ceramide and plasma cholesterol levels.^45^ In conclusion, GLP‐1 RAs may contribute to the reduction of ceramide levels by improving Acer2 expression.

In this study, we analyzed the correlation between ceramide and *Lactobacillus* and briefly studied the changes of Acer2 after treatment with *L. reuteri*. Although the data suggests that Acer2 may be related to the GLP‐1 RA‐induced ceramide metabolism, further evidence is needed to determine whether these changes in ceramides and Acer2 expression are responsible for the fat browning/thermogenesis observed after GLP‐1 RA treatment in mice. Additionally, whether the Acer2‐mediated signal is also responsible for the anti‐obesity effect of *L. reuteri* in mice needs to be further explored.

In this study, we found that GLP‐1 RA reduces body weight in HFD mice by the following mechanisms: (1) GLP‐1 RA increases fat browning and thermogenesis; (2) after GLP‐1 RA treatment, the gut microbiota structure and composition of mice were changed, and the probiotics represented by *L. reuteri* were enriched; (3) GLP‐1 RA treatment altered lipid metabolism in mice, especially sphingolipids, including reduced serum ceramide and sphingomyelin levels. In addition, we also found that *L. reuteri* combined with (Ex‐4)_2_‐Fc had a synergistic effect on weight reduction and metabolism improvement in HFD mice, which may provide ideas for the clinical use of gut microbiota.

## 4 MATERIALS AND METHODS

### 4.1 Animals and diets

Male C57/6j mice (6–8 weeks old) were purchased from Vital River and singly housed under standard laboratory conditions (12/12 h light/day cycle, 22–24°C, 40%–60% humidity) with free access to food and water. The control group (NS) consisted of five mice that were given a normal diet and distilled water. The other mice were fed a high‐fat diet (D12492, OpenSource Diets). After 8 weeks on the high‐fat diet, mice were randomly selected and assigned to four groups. Mice were given (Ex‐4)_2_‐Fc treatment (1.8 mg/kg, every 6 days, i.p.) and were gavaged daily with *L. reuteri* (10^9^ CFU). The gavage treatment lasted for 14 days. Body weight and food intake were monitored every day. Epididymis adipose tissue from the epididymis and visceral adipose tissue and BAT from the interscapular region were separated and rinsed with PBS and then weighed immediately. Fresh fecal samples of all mice were collected. To minimize possible circadian effects, all collected samples were stored at −80°C for further analysis.

### 4.2 *L. reuteri* culture

*L. reuteri* was grown in Man Rogosa Sharpe (MRS, ELITE‐MEDIA) at 37°C for 16 h, and the concentration was detected with a microplate reader. The bacteria were harvested using centrifugation (2000 rpm, 5 min), washed twice with PBS, and resuspended in MRS.

### 4.3 Glucose tolerance test

For the GTT, mice were fasted for 16 h on the 14th day after (Ex‐4)_2_‐Fc treatment and intraperitoneally injected with D‐glucose in sterile water (2 g/kg). Then, blood samples were collected from the tip of the tail vein at 0, 30, 60, and 120 min after injection for measurement of blood glucose using a Roche glucometer.

### 4.4 Untargeted metabolomics study

Blood samples were collected through eye vessels by removing mouse eyeballs on the 14th day and then centrifuged at 4°C at 5000 rpm for 10 min to isolate serum supernatant for serum metabolomic analysis. Serum samples were stored at −80°C and analyzed at Novogene.

### 4.5 Fecal DNA extraction and 16S rDNA amplicon sequencing

Fecal DNA was extracted using the Stool DNA Isolation Kit (FOREGENE) according to the manufacturer's instructions. Purified fecal DNA of 1 ng was used for PCR amplification. 16S/18S rRNA genes were amplified using specific primers with barcodes. Thermal cycling consisted of initial denaturation at 98°C for 1 min, followed by 30 cycles of denaturation at 98°C for 10 s, annealing at 50°C for 30 s, and elongation at 72°C for 30 s. Finally, 72°C was used for 5 min. The PCR products were then sequenced on an Illumina HiSeq sequencer at Novogene.

### 4.6 RNA extraction and qPCR

RNA was isolated from tissues by using an Animal Total RNA Isolation kit (FOREGENE), and 500 ng of RNA from each sample was reverse‐transcribed using HiScript RT supermix for qPCR (Vazyme) according to the manufacturer's instructions. mRNA and DNA expressions were quantified by RT‐PCR with SYBR green PCR master mix (Vazyme). The sequences of the gene‐specific primers (TSINGKE) are shown in Table S1. β‐Actin was used as an internal control for normalizing the mRNA levels of the tested genes.

### 4.7 Serum insulin measurement

Serum insulin was measured by a mouse insulin ELISA kit (EZRMI‐1, Millipore). The precoated 96‐well plate was removed from the kit, and when it returned to room temperature, the plate was washed three times with washing buffer. Finally, the residual liquid in the well was patted dry. Detection buffer of 10 μL, matrix solution, and sample were added to the corresponding wells. Detection antibody was added to each well, placed on a shaking table (400–500 rpm), and incubated for 2 h at room temperature. The cells were washed three times by adding washing buffer. Then, enzyme solution was added for 30 min. The cells were washed six times, chromogenic agent was added, and color was developed at room temperature for 20 min. Another termination solution was added, and the OD450 value was read on a microplate reader.

### 4.8 Histological analysis

Fresh liver and adipose tissue were fixed in 4% (v/v) formaldehyde solution for 48 h, embedded in paraffin wax, sectioned, and stained with H&E. UCP‐1 antibody (EPR20381, Abcam) was used for immunohistochemical staining. For oil red O staining, after liver tissue was removed, it was frozen in liquid nitrogen after embedding in an embedding agent, and frozen sections were obtained with a thickness of approximately 5 μm. Histological images were obtained using an optical microscope (Nikon).

### 4.9 Statistical analyses

Unless otherwise specified, statistical analyses were performed with the two‐sided Student *t* test (two groups) and one‐way ANOVA (more than two groups), followed by Tukey's multiple comparisons test, where appropriate, using GraphPad Prism software. Data are depicted as the means ± SEMs; a *p* value <0.05 was considered to be statistically significant.

## AUTHOR CONTRIBUTIONS

Li Yang supervised and supported the study. Ke Lin gathered all the data, drew all the figures, and composed the manuscript. Chunyan Dong helped with the lipid metabolomics experiments and the detection and analysis of gut microbiota. Binyan Zhao helped with *L. reuteri* culture and other experiments. Bailing Zhou helped with the data analysis and the design of the study. All authors have read and approved the final manuscript.

## CONFLICT OF INTEREST STATEMENT

The authors have no conflicts of interest to declare.

## ETHICS STATEMENT

All protocols in this study were approved by the experimental animal ethics committee on state key laboratory of Biotherapy, Sichuan University (approval number: 20190923028).

## Supporting information

Supporting Information

## Data Availability

Raw data can be made available from corresponding author.
