# Supplementary material for: Glucagon‐like peptide‐1 receptor agonist regulates fat browning by altering the gut microbiota and ceramide metabolism
Source: MedComm (2020). 2023 Nov 20;4(6):e416. doi: 10.1002/mco2.416 (PMC10661313; doi:10.1002/mco2.416)
Supplement: Supplementary file 1 — Supporting Information [file MCO2-4-e416-s001.docx]

**Glucagon-like Peptide-1 Receptor Agonist Regulates Fat Browning by Altering the Gut Microbiota and Ceramide Metabolism**

Ke Lin^#^, Chunyan Dong^#^, Binyan Zhao, Bailing Zhou and Li Yang

#These authors have contributed equally to this work

Department of Biotherapy，Cancer Center and State Key Laboratory of Biotherapy, West China Hospital, Sichuan University, Chengdu, 610041, China.

Correspondence: Li Yang, Department of Biotherapy，Cancer Center and State Key Laboratory of Biotherapy，West China Hospital, Sichuan University, Chengdu, 610041, China. Emial: yl.tracy73@gmail.com

**Figure S1**


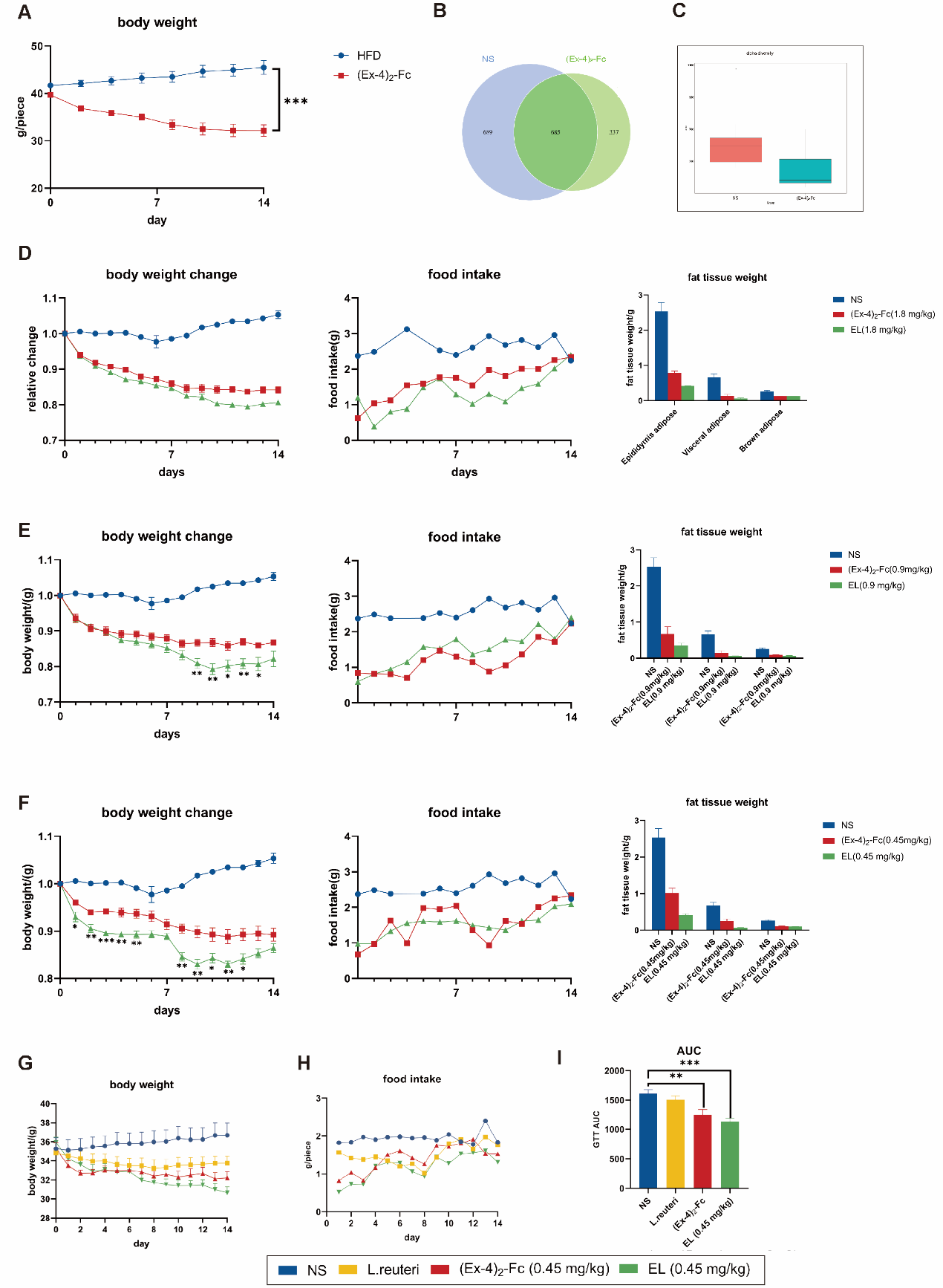


**S1: A**: Body weight in NS group and (Ex-4)_2_-Fc group. **B**: Venn diagram, representing the number of common and unique gut microbiota species in NS group and (Ex-4)_2_-Fc group. **C**: Beta diversity refers to the difference in species composition between communities of different habitats along the environmental gradient or the rate of species turnover along the environmental gradient, i.e. the difference in species composition between communities of different sites. **D**: Body weight change, food intake and fat tissue weight of treatment with 1.8 mg/kg (Ex-4)_2_-Fc and *L.reuteri*. **E**: Body weight change, food intake and fat tissue weight of treatment with 0.9 mg/kg (Ex-4)_2_-Fc and *L.reuteri*. **F**: Body weight change, food intake and fat tissue weight of treatment with 0.45mg/kg (Ex-4)_2_-Fc and *L.reuteri*. **G**: Body weight in NS, L.reuteri, (Ex-4)_2_-Fc(0.45 mg/kg) and EL(0.45 mg/kg) group. **H**: Food intake of treatment with 0.45 mg/kg (Ex-4)_2_-Fc and *L,reuteri*. **I**: AUC of GTT. Data are shown as the mean ± S.E.M. *P<0.05 vs NS group.

**Figure S2**


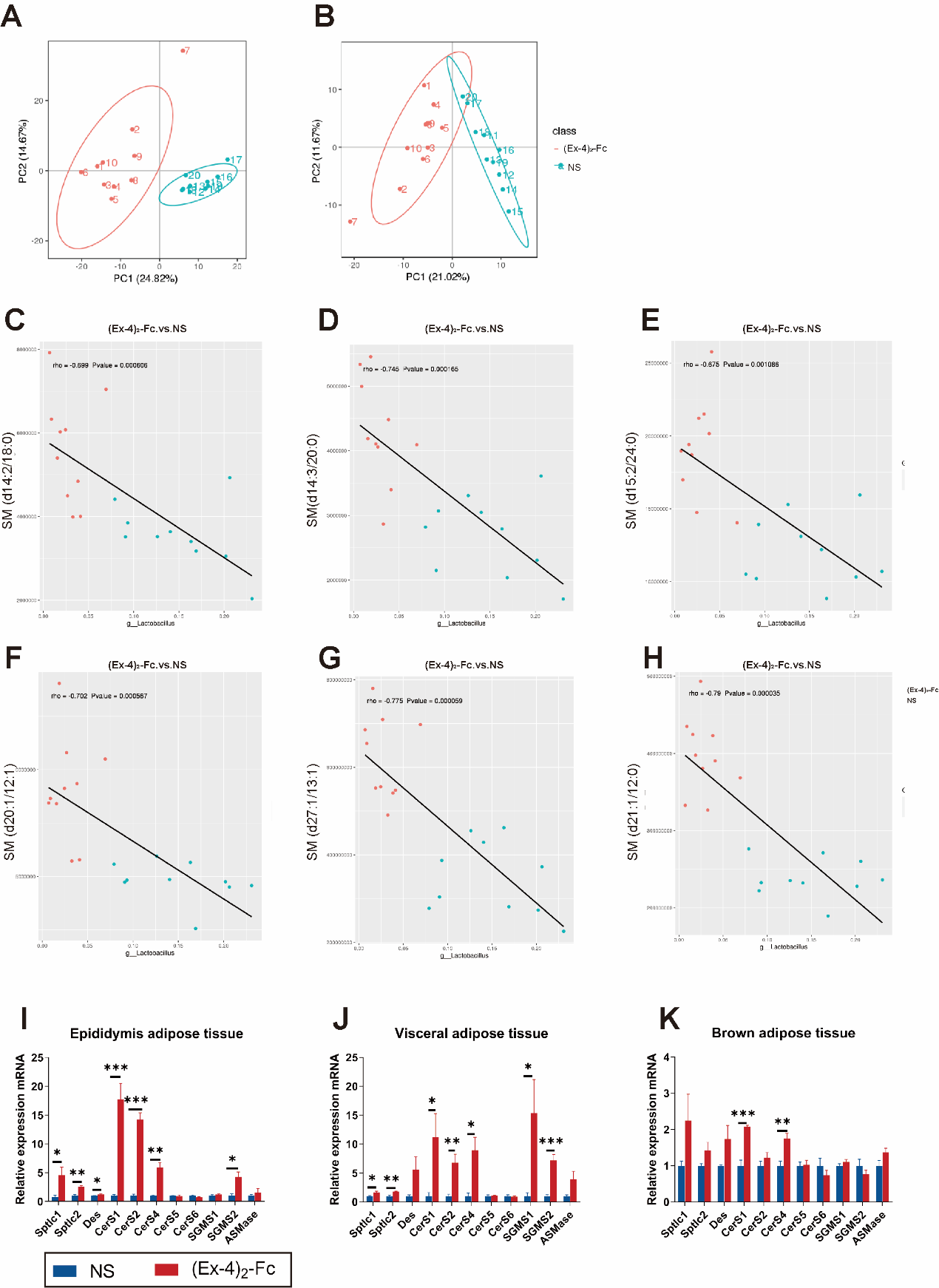


S2. . **A**: PCA diagram of non-targeted positive ions metabolomics. **B**: PCA diagram of non-targeted negative ions metabolomics. **C-H**: The scatter plot of Sphingomyelin correlation with Lactobacillus. **I-K**: Ceramide synthetase of epididymis adipose tissue, visceral adipose tissue and brown adipose tissue.

**Tables 1**

**Table 1. primers in qpcr**

| Primer name | Base sequence (5'to 3') |
| --- | --- |
| Mus β-actin-Forward | GAT GTA TGA AGG CTT TGG TC |
| Mus β-anctin-Reverse | TGT GCA CTT TTA TTG GTC TC |
| Mus ucp1-Forward | ACT GCC ACA CCT CCA GTC ATT |
| Mus ucp1-Reverse | CTT TGC CTC ACT CAG GAT TGG |
| Mus dio2-Forward | CAG TGT GGT GCA CGT CTC CAA TC |
| Mus dio2-Reverse | TGA ACC AAA GTT GAC CAC CAG |
| Mus Prdm16-Forward | CAG CAC GGT GAA GCC ATT C |
| Mus Prdm16-Reverse | GCG TGC ATC CGC TTG TG |
| Mus Pgc1a-Forward | CCC TGC CAT TGT TAA GAC C |
| Mus Pgc1a-Reverse | TGC TGC TGT TCC TGT TTT C |
| Mus Pgc1b-Forward | TCC TGT AAA AGC CCG GAG TAT |
| Mus Pgc1b-Reverse | GCT CTG GTA GGG GCA GTG A |
| Mus Cidea-Forward | GCC GTG TTA AGG AAT CTG CTG |
| Mus Cidea-Reverse | TGC TCT TCT GTA TCG CCC AGT |
| Mus Cox8b-Forward | GAA CCA TGA AGC CAA CGA CT |
| Mus Cox8b-Reverse | GCG AAG TTC ACA GTG GTT CC |
| Mus Cox7a-Forward | CAG CGT CAT GGT CAG TCT GT |
| Mus Cox7a-Reverse | AGA AAA CCG TGT GGC AGA GA |
| Mus Cpt1a-Forward | ACG TTG GAC GAT CGG AAC A |
| Mus Cpt1a-Reverse | GGT GGC CAT GAC ATA CTC CC |
| Mus Cd137-Forward | CGT GCA GAA CTC CTG TGA TAA C |
| Mus Cd137-Reverse | GTC CAC CTA TGC TGG AGA AGG |
| Mus Tbx1-Forward | GGC AGG CAG ACG AAT GTT C |
| Mus Tbx1-Reverse | TTG TCA TCT ACG GGC ACA AAG |
| Mus Tmem26-Forward | TGT TTG GTG GAG TCC TAA GGT C |
| Mus Tmem26-Reverse | ACC CTG TCA TCC CAC AGA G |
| Mus SGMS1-Forward | AAG ATA GCC ACC GAC TCC GAA GG |
| Mus SGMS1-Reverse | ACT GTC CGT TCT GTC CAC TCT CC |
| Mus SGMS2-Forward | TGG TAG GCA GCA GTA GTC GTA GAG |
| Mus SGMS2-Reverse | GAC AAG AAC ATC CAC GGC TCA GG |
| Mus SPT-Forward | CCAGCAGGCACCACCACAAC |
| Mus SPT-Reverse | CCA GAC AAG GCA GAG AAG ACA GAC |
| Mus CerS1-Forward | CCG CCT TTA ATC CCA GCA CTC AG |
| Mus CerS1-Reverse | TCC TCC TCC TCC TCT TCC TCT CC |
| Mus CerS2-Forward | AGT GGA GCA GTA GCG GAC CTT C |
| Mus CerS2-Reverse | AGT AGC AGC AAC AGC AGC AAC AG |
| Mus CerS4-Forward | GAG GCT GGC GTT GCT GGA ATA G |
| Mus CerS4-Reverse | TCC AAC TCC TCC TCT GCT CTT GAG |
| Mus CerS5-Forward | TGG CCA ATT ATG CCA GAC GTG AG |
| Mus CerS5-Reverse | GGT AGG GCC CAA TAA TCT CCC AGC |
| Mus CerS6-Forward | GCA TTC AAC GCT GGT TTC GAC |
| Mus CerS6-Reverse | TTC AAG AAC CGGB ACT CCG TAG |
| Mus ASMase-Forward | CAA CGC AGC AGG AGG CAC AC |
| Mus ASMase-Reverse | GGC AGG CAT CGC ATC TGG AAG |
| Mus DEGS-Forward | TCC CTC AGT CTG TGT CAC CCA TC |
| Mus DEGS-Reverse | CTCACTTGCGATCTTCCTCACCTG |
| Mus sptlc1-Forward | AGG GTT CTA TGG CAC ATT TGA TG |
| Mus sptlc1-Reverse | TGG CTT CTT CGG TCT TCA TAA AC |
| Mus sptlc2-Forward | CAA AGA GCT TCG GTG CTT CAG |
| Mus sptlc2-Reverse | GAA TGT GTG CGC AGG TAG TCT ATC |
| Mus Acer1-Forward | TCT GAG GTG GAT TGG TGT GAG |
| Mus Acer1-Reverse | TGA GGG GTC CAA AGA TGA GGA |
| Mus Acer2-Forward | TGT GGC ATA TTC TCA TCT GCC T |
| Mus Acer2-Reverse | CAA TAA AAG CCC ATT TCT CGC TG |
| Mus Acer3-Forward | TGT GAT TCA CTG AGG AAC TTT CG |
| Mus Acer3-Reverse | AGA AAC TTC ACT TTT GGC CTG TA |
